# Effects of Alfaxalone or Propofol on Giant-Breed Dog Neonates Viability During Elective Caesarean Sections

**DOI:** 10.3390/ani9110962

**Published:** 2019-11-13

**Authors:** Monica Melandri, Salvatore Alonge, Tanja Peric, Barbara Bolis, Maria C Veronesi

**Affiliations:** 1 Società Veterinaria “Il Melograno” Srl, 21018 Sesto Calende, Varese, Italy; drmonicamelandri@gmail.com; 2 Department of Agricultural, Food, Environmental and Animal Sciences, University of Udine, 33100 Udine, Italy; tanja.peric@uniud.it; 3 Department of Veterinary Medicine, Università degli Studi di Milano, 20100 Milan, Italy; barbarabolis.medicoveterinario@gmail.com (B.B.); maria.veronesi@unimi.it (M.C.V.)

**Keywords:** alfaxalone, Apgar score, canine neonates, cortisol, C-section, fetal fluids

## Abstract

**Simple Summary:**

Nowadays, thanks to the increased awareness of owners and breeders and to the most recent techniques available to veterinarians, the management of parturition, especially of C-sections, has become a topic of greater importance. Anesthesia is crucial and must be targeted to both the mother and neonates. The present study aimed to evaluate the effect of the induction agent alfaxalone on the vitality of puppies born from elective C-section, in comparison to propofol. After inducing general anesthesia for elective C-section, puppies from the mothers induced with alfaxalone had higher 5-min Apgar scores than those induced with propofol. The concentration of cortisol in fetal fluids collected at birth is neither influenced by the anesthetic protocol used, nor does it differ between amniotic and allantoic fluids. Nevertheless, the cortisol concentration in fetal fluids affects the relationship between anesthesia and the Apgar score: the present study highlights a significant relationship between the anesthetic protocol used and Apgar score in puppies, and fetal fluids cortisol concentration acts as a covariate of this relationship. Alfaxalone is a safe and effective drug for the induction of anesthesia in dogs, and it can be successfully employed for elective C-section, with neonatal wellbeing improvements.

**Abstract:**

Attention must be paid to C-section anesthesia effects on mother and offspring. Alfaxalone induction results in improved puppy viability when compared to propofol. The present study aims to evaluate effects of alfaxalone or propofol induction for elective C-section on newborns, expressed as Apgar score and fetal fluids cortisol concentration. Anesthesia was induced with alfaxalone 3 mg/kg iv in 5 bitches (group A), and propofol 4 mg/kg iv in another 5 (group P), maintained with isoflurane. Amniotic and allantoic fluids were collected to determine cortisol concentration. Apgar score, litter size, newborn gender, birth-weight, maternal age, and parity were recorded. ANOVA, U Mann-Whitney test and ANCOVA assessed the effects of drugs on the Apgar score and fetal fluids cortisol. Thirty-six puppies were randomly selected for the study: 16 from group A and 20 from group P. Only the Apgar score significantly differed between groups. ANCOVA confirmed a significantly higher Apgar score in group A underlining the influence of fetal fluids cortisol concentrations, both resulting in covariates. Present results confirm the effect of anesthesia on the Apgar score of newborns, which is significantly higher for alfaxalone than propofol, suggesting the use of fetal fluids cortisol as a covariate. These findings could be a starting point for further investigations when less viable puppies are detected or expected, such as during an emergency C-section.

## 1. Introduction

In dogs, as in other species, at Caesarean sections (C-sections) that are either planned or occur in an emergency, particular attention must be paid to anesthesia effects on both the mother and the offspring. On the maternal side, the so-called “gestational anti-nociception” must be taken into account [1,2,3], allowing reduced drug dosages by 25% [4], and decreasing the isoflurane mean alveolar concentration (MAC) by 28%/40% [5,6]. The latter is reported in ewes [5] and humans [6], but it can be reasonably extended to other species, including dogs. This aspect is particularly relevant since, on the offspring side, many anesthetic agents cross the placenta and the fetal blood-brain barrier, leading to a variable extent of neurological and cardio-respiratory depression that could impair newborn viability [4].

At term, pregnant dogs need a 5-min pre-oxygenation to face the reduction of the residual pulmonary volume [7]. Moreover, even if the existence of the aorto-caval syndrome was never demonstrated in dogs [8,9,10], a supplementary fluid therapy must be given to face the potential systemic hypotension, resulting from the extraction of the uterus from the abdomen, in a so called “vacuum phenomenon” [11].

A standardized anesthetic protocol for C-section does not exist in dogs. Current practice in anesthesia prefers to assess each patient and come up with individual protocols based on patient presentation, history, procedure, and physical examination. Moreover C-section anesthesia implies several specific prerequisites to be satisfied: maneuverability, minimum cardio-respiratory depression, total reversibility of side effects on neonates, long-lasting analgesia, fast and safe maternal awakening, and suitable care of the offspring [12,13].

The impact of anesthesia at C-section on outcomes for puppies is an important issue in canine reproduction. In the last years, several studies have been performed to assess the best protocol aimed to reduce fetal distress and maximize neonatal outcome [13,14,15,16]. However, the study of the possible effect of a drug on fetal distress and neonatal outcome could be affected by several factors, namely the methods to assess fetal distress or newborn viability, the clinical requirement of C-section (emergency or planned), and the type of anesthesia protocol [13,14,15,16]. Furthermore, also the health and the management of the pregnant bitch could influence the fetal well-being and development [17,18], and, when an elective C-section is planned, also the identification of the correct day for surgery, in terms of physiologic end of pregnancy and fetal maturation for birth is pivotal [18,19,20,21,22,23,24].

At a C-section, thyopental, ketamine, xylazine, and methoxyflurane should definitely be avoided [25,26].

Propofol induction followed by isoflurane maintenance has positive effects on neonatal survival at 7 days [27]. Propofol is a sedative-hypnotic agent, which induces depression by enhancing the effects of the inhibitory neurotransmitter gamma-aminobutyric acid (GABA) [28]. Chemically, it is represented by a phenolic compound, only slightly soluble in water, which is marketed as an aqueous emulsion [29]. This mixture contains, among other ingredients, egg lecithin, which makes propofol able to support microbial growth and endotoxin production, thus this product must be handled carefully [29]. Propofol has a rapid onset of action, thanks to its fast uptake in the central nervous system, a large volume of distribution, by its highly lipophilic nature, and a quite fast disappearance from plasma, via hepatic metabolism, primarily based on conjugation pathways, and mainly urinary excretion [30]. Propofol decreases intracranial and cerebral perfusion pressure, and, as a result of arterial and venous vasodilation, it transiently decreases arterial pressure and myocardial contractility [31]. Patients induced in general anesthesia with propofol can experience a brief period of apnea, followed by a short phase of hypercapnia, particularly if the intravenous administration occurs too fast [32].

In 2004, Luna and colleagues have demonstrated that the optimal procedure for anesthesia at C-section is represented by a loco-regional epidural technique, but it has technical drawbacks of undesired prolonged paralysis, hypo-tension, and restlessness, that may lead to the induction of general anesthesia [33].

Alfaxalone, a steroidal molecule with GABA-agonist action that was recently introduced on the Italian veterinary market, shows many features, making it particularly suitable for small animal anesthesia in the obstetrical field, especially for C-sections. Alfaxalone leads to a quick and smooth induction of general anesthesia, with a subsequent rapid recovery and minimum respiratory depression; it is characterized by a wide maneuverability, fast clearance, and short plasmatic half-life [34,35]. At the end of the twentieth century, alfaxalone was commonly used, especially in feline medicine, in a commercial formulation known as “saffan”, a mixture of alfaxalone and alfadolone solubilized by an oily cromophore [36]. The latter was responsible for severe anaphylactic reactions in dogs, mediated by a huge histamine release, which limited the use of the drug in clinical practice, with the constant necessity of a premedication with anti-histaminic agents [37]. Nevertheless, already in 1981, Bomzon reported the use of alfaxalone in bitches undergoing C-section, describing it as a safe and predictable inductor for general anesthesia, with higher properties if compared to thyopental anesthesia [38]. Subsequently, a new formulation of alfaxalone was introduced on the market, dedicated to small animal anesthesia, thanks to a new solubilizing agent, represented by cyclodextrines, not inducing hystamine release in the dog [35,39].

In dogs, anesthesia induction with alfaxalone at emergency C-section was recently related to higher newborn viability, assessed by Apgar score, in comparison to propofol [14]. Anesthesia induction with alfaxalone at elective and emergency C-section was also qualified as equivalent to propofol in terms of neonatal vitality [15], but in this case the evaluation of puppies was less objective as it was assessed scoring straightening, suction, ano-genital, and flexion reflexes. Finally, anesthesia maintenance by alfaxalone in continuous rate infusion (CRI) was attempted at elective C-section, but its results were inadvisable in clinical practice compared to isoflurane, because it lowers the neonatal Apgar score and maternal recovery quality, and it requires repeated rescue boluses and high total dosages of alfaxalone, implying slow procedures [16]. The lighter depth of anesthesia obtained by alfaxalone CRI was also supposed to be responsible for a higher arterial blood pressure [16].

Concerning the objective evaluation of the impact of anesthesia on puppies, veterinary neonatologists have to face the limited possibilities for blood-gas analysis or other investigations that could harm the health of small and fragile newborn puppies. Recently, the Apgar score, and the cortisol concentration in amniotic and allantoic fluids were suggested as neonatal parameters possibly affected by the delivery management [17,40,41,42]. The Apgar score evaluation, introduced 10 years ago by Veronesi and colleagues [40,41], provides a simple and useful tool for the evaluation of canine newborns. In particular, it represents the easiest tool for estimating neonatal viability [40,41]; thus, its evaluation allowed a comparison of the effect of different drugs used at C-sections in some studies [14,16]. On the other hand, thanks to their easy and safe collection at C-sections, fetal fluids have been reported as being useful for newborn puppies evaluation at birth, too [42,43,44]. Among fetal fluids compounds, amniotic and allantoic cortisol, as the final products of the hypothalamus-pituitary-adrenal (HPA) axis activation, have been reported as possible markers for newborn assessments and to detect those puppies requiring special surveillance in the first 24 h of life [42]. Therefore, since both parameters were related to the short-term survival of newborn dogs, fetal fluids cortisol concentrations analysis, together with the Apgar score assessment, provides a tool for the evaluation of canine newborns [40,41,42].

The aim of the present study was to evaluate the effect of alfaxalone or propofol, used to induce general anesthesia for elective C-section in giant-breed dogs, on newborn viability, measuring the neonatal Apgar score and fetal fluids cortisol concentration.

## 2. Materials and Methods

### 2.1. Ethics

The study was performed in accordance with the animal welfare committee ethical guidelines and all procedures were carried according to the Italian legislation on animal care (DL 116, 27/01/1992) and the European Guidelines on Animal Welfare (Directive 2010/63/EU). A written informed owner consent was obtained, not only to submit pregnant dogs to elective C-section, but also for the collection of clinical records and fetal fluids for research purposes.

### 2.2. Animals

Ten giant-size purebred bitches (53–75 kg body-weight; 2–6 years of age; 1–3 parity) belonging to four breeds (Great Dane, Newfoundland, Maremmano, Saint Bernard) were enrolled. All sourced animals were client owned, and housed in the respective kennels. All bitches were proven to be healthy at the breeding soundness examination (time of patients recruitment) performed before the beginning of pregnancy in order to avoid the effect of maternal illness on perinatal health [45,46]. The breeding soundness examination included the clinical history of the bitch, a physical examination (general objective examination, combined with an objective examination of the reproductive organs), a haemochromocytometric exam, a biochemical profile, and an ultrasonographic exam of the whole reproductive tract. They all were fed with the same commercial diet according to metabolic requirements for gestation, according to veterinary suggestions. No remarkable differences existed in the pregnancy management that could artificially enhance different neonatal viability and maternal or fetal stress in any of the groups. Only pregnancies without complications, showing normal fetal development, as assessed by fetal biometry, were kept in the study [17,19,20]. Because of previous history or prediction of troubles at parturition, C-section was, in all cases, planned for the health of mothers and puppies.

### 2.3. Procedure

Because of the importance of ensuring the correct matching between the calculated day of an elective C-section and the actual term of pregnancy in relation to fetal preparation for birth [17], the elective C-section was planned on the basis of several cumulative parameters. They were represented by blood progesterone concentrations (MiniVidas, BioMerieux, Marcy l’Etoile, France) measured during heat [19], prediction of parturition day, estimated by fetal biometry [20], and the confirmation of approaching parturition by blood progesterone concentrations < 2 ng/mL [18]. Also, a clinical monitoring of fetal well-being [47] and maternal impending parturition were daily assessed in the last 3-5 days preceding the expected date of parturition.

On the day of the C-section, the bitches were fasted for 12 h before surgery. [11]. No pre-medication was given; an intravenous catheter was placed; 5-min pre-oxigenation and fluid therapy with ringer lactate 5 mL/kg/h iv (Ringer Lattato, BBraun Milano Spa, Milano, Italy) were administered. Concerning anesthesia, bitches were randomly assigned to alfaxalone (Alfaxan, Dechra Veterinary Products Srl, Torino, Italy) 3 mg/kg iv (group A), or propofol (Proposure, Merial, Lyon, France) 4 mg/kg iv (group P) anesthetic induction. Both drugs were administered via the iv catheter titrated to effect to reach oro-tracheal intubation. Anesthesia was then maintained with isoflurane 2% (Vetflurane, Virbac, Milan, Italy) in oxygen 90%–95%, delivered via an oxygen concentrator (Nuvo Mark 8, GCE Mediline, Malmo, Sweden). An open circuit Mapleson type C was used. Isoflurane dosage was always verified as expired isoflurane; its requirement was clinically assessed in order to maintain a surgical anesthesia classified as stage III plane 2 [48]. Opioids (methadone 0.2mg/kg im, Semfortan, Dechra Veterinary Products Srl, Torino, Italy) and NSAIDs (meloxicam 0.2mg/kg im, Inflacam, Virbac, Milan, Italy) were administered only after the extraction of the last puppy.

The operating team always involved the same veterinarians: two surgeons, an anesthetist, two neonatologists, and one person exclusively dedicated to fetal fluids collection. Surgeons, neonatologists, and the person entrusted to collect fetal fluids were blind to the inductor agent used by the anesthetist. The anesthetist randomly assigned bitches to group A or group P: the casual randomization assigned bitches with odds enrolment number to group A and bitches with even enrolment number to group P. The two neonatologists were of equal expertise and followed the same standardized procedures in the assistance and clinical evaluation of puppies.

Amniotic and allantoic fluids were aseptically collected at fetal bags opening. Fluids were immediately centrifuged at 1000 rpm for 10 min, supernatant was removed, and samples were frozen at −20 °C to be later analyzed by Radio-Immuno Assay (RIA) for cortisol concentrations (Cortisol, [1,2,6,7-3H (N)] PerkinElmer Life Sciences, Boston, MA, USA) by Top-Count (PerkinElmer Life Sciences, Boston, MA, USA). Analysis was always performed within 3 months from fluid collection [42,43]. The cortisol analysis in amniotic and allantoic fluids was performed following the procedure described by Bolis and colleagues [42].

Sixty-nine healthy, viable [21,22,40], and normal-weighted [23,49,50] puppies were born. In order to ensure the collection of both amniotic and allantoic fluids from the same puppy, and to reduce to the minimum the possibility of mistakes between fetal fluids collection/identification and newborn records, it was chosen not to sample entire litters, but around half of the total number of puppies from each litter. Thus, among the sixty-nine newborns, thirty-six puppies were sampled: 16 from group A bitches and 20 from group P bitches. Indicatively, every second puppy was sampled, allowing an equal representation of puppies extracted at the beginning, in the middle, and at the end of the C-section. The order of puppies at delivery was recorded, and no specific differences were appreciated. A potential bias concerning puppies delivered last, which would be subjected to isoflurane for a longer period than puppies delivered earlier, possibly affecting Apgar scores and cortisol concentrations, could be suspected. However, in the present study, it would be present in both alfaxalone and propofol groups, thus it does not affect the statistical comparison between the two groups. Moreover, groups A and P were statistically proven not to be different concerning epidemiological parameters, including the litter size.

Apgar score was assessed in all puppies 5 min after birth, and puppies were defined as viable when the Apgar score was ≥7 [40].

Litter size, newborn gender and birth-weight, and maternal age and parity were also recorded.

### 2.4. Statistical Analysis

All obtained data were reported on Excel 2010 Office files, and mean values ± SD were calculated for each parameter. As Apgar score is a non-parametric ordinal variable, it was expressed as median value, accompanied by assumed minimum and maximum values. All data were tested for normal distribution by a Shapiro-Wilk test. The statistical analysis was performed by ANOVA, U Mann-Whitney test and ANCOVA, to assess the effect of the two anesthetic drugs on Apgar score and fetal fluids cortisol concentration, as well as the possible effect played by other maternal or neonatal parameters.

First of all, the ANOVA was used to compare enrolled litters and puppies numbers, male to female ratio, birth-weight, litter size, maternal age and parity between the two study groups, in order to verify the absence of any epidemiological difference between group A and P that, if present, could have artificially biased results of the present study. Then, ANOVA was also used to compare cortisol concentrations in amniotic and allantoic fluids within both groups, in order to verify similarities in their trends. Finally, ANOVA was used to compare amniotic cortisol and allantoic cortisol concentrations, while the U Mann-Whitney test was applied for Apgar scores, between the two study groups, in order to verify the possible effects of the two induction protocols.

Furthermore, the possible effect played by other variables on the relationship existing between the anesthetic induction protocol and Apgar score was assessed by ANCOVA, in which the type of anesthetic induction protocol (alfaxalone or propofol) was considered as the independent variable and the Apgar score as the dependent variable. Amniotic and allantoic cortisol concentrations and epidemiological parameters (male to female ratio, birth-weight, litter size, maternal age and parity) were evaluated as concomitant variables (*alias* possible covariates). The ANCOVA was chosen because it is useful when the analysis of data of a certain variable is possibly associated to a covariate preventing the creation of homogeneous groups to compare. In the present study, in order to evaluate the real effect of anesthetic protocol induction on Apgar score, the cortisol level of amniotic and allantoic fluids and epidemiological data could represent possible confounding factors, leading to biased results. Theoretically, this problem could have been bypassed by forming homogeneous experimental groups for each possible confounding covariate. This was not practically possible and even not so useful, since the covariates were factors that could be measured. Moreover, since possible covariates taken into account in the present study were continuous variables, it was more useful to use them as such and not as limited intervals, which would not reflect the actual normal heterogeneity in concomitant variables levels, allowing us to determine the effect of the induction protocol only in those animals with a specific level of the concomitant variables. Thus, the results would have not been generalizable and of scarce relevance in clinical practice. Such an approach reduced the error variance due to heterogeneity and corrected mean values of groups to obtain a correct estimate of the anesthesia induction effect on the Apgar score.

Results were considered significant for *p* < 0.05. The statistical analysis was performed with the online tools VassarStats: Website for Statistical Computation (http://vassarstats.net, Vassar College, New York, NY, USA) and Social Science Statistics (https://www.socscistatistics.com, Jeremy Stangroom, USA).

## 3. Results

Sixty-nine healthy, viable, and normal-weighted puppies were born. Among them, thirty-six puppies were sampled: 16 from group A bitches and 20 from group P bitches. They all showed a subsequent normal growth for the respective breeds, with no deaths reported in the first year of life.

The ANOVA statistical analysis did not show significant differences in cortisol concentrations between amniotic and allantoic fluids, neither within nor between groups A and P (Table 1).

Similarly, clinical records, such as enrolled litters and puppies numbers, the male to female ratio, birth-weight, maternal age and parity, and litter-size did not show statistically significant differences between the two groups.

Conversely, the 5-min Apgar score was statistically (*p* <0.02) higher in group A than in group P (Table 2).

Finally, the ANCOVA confirmed the effect of anesthesia induction protocol on Apgar score, significantly higher in group A than in group P (*p* < 0.05). Furthermore, it highlighted the role of both amniotic and allantoic fluids cortisol concentrations as covariates on the relationship existing between anesthesia induction protocol and 5-min Apgar score (*p* = 0.01 and *p* = 0.004, respectively). No covariance effect was found concerning either maternal or neonatal epidemiological data.

## 4. Discussion

When elective C-section in dogs is required, the most important issue is the correct identification of the date for surgery, managing to perform it when gestation reaches its real physiological end and fetal maturity for birth [17]. This goal can be achieved only when multiple parameters are considered together. For this reason, in the present study, blood progesterone concentration during heat [19] was matched to fetal biometry formulae for the prevision of the day of expected parturition, to verify the normal fetal development [20,24]. Finally, in order to increase precision of the estimation, blood progesterone concentration decrease to pre-parturient values was assessed [18], while clinical and ultrasonographic maternal preparation for birth was checked [47], as well as the fetal maturation of some organs [22].

Therefore, in the present study, aimed to evaluate the comparison between two different protocols for anesthesia induction on neonatal outcome at elective C-section, the date of surgery was scrupulously identified, and Apgar score and cortisol concentration in both fetal fluids were assessed.

The U Mann-Whitney test showed a significantly higher Apgar score when anesthesia induction was performed with alfaxalone rather than with propofol. Moreover, the ANCOVA suggested the role of fetal fluids cortisol as covariate. This means that, allocating the same fluid cortisol concentration to all puppies, the pharmacological effect on Apgar score would be even more significant. Under a statistical point of view, detecting the heterogeneity factor represented by cortisol, the error variance gets reduced and the positive effect of alfaxalone on puppy viability in terms of the Apgar score is more markedly underlined. This result must be interpreted cautiously. In fact, the statistically significant difference was found between puppies scored as median value 10 (minimum 10, maximum 10) in the A group vs. median value 9 (minimum 8, maximum 10) in P group, which means puppies equally classified in the highest score of viability [40,41]. Even if the clinical relevance of this finding is restrained, present results could be a starting point for further investigations in situations with diagnosis or expectation of less viable puppies, such as at emergency C-section. In this setting, the use of alfaxalone for anesthesia induction might be better than other protocols, because cortisol is supposed to be higher due to the hyper-activation of the HPA-axis of both mother and fetuses [51], and diverse anesthetic drugs might play a more pronounced role on newborn viability. In fact, the effect of cortisol concentration in fetal fluids on the association between alfaxalone and higher Apgar scores seems to suggest that the effect of alfaxalone on newborns better viability could be related to the fetal HPA-axis activation [51]. However, based on actual knowledge about canine fetal maturation, it is not possible to link this finding to puppies maturity. As such, it must be remarked that, in the present study, all C-sections were planned before the beginning of spontaneous parturition, but as close as possible to the physiologic term of pregnancy. At this time, fetuses are mature enough to adapt to extra-uterine life and to survive after birth. In fact, apart from viability assessment by the Apgar score, all the puppies were completely developed [44], healthy [22], and normal-weighted [23,49,50], and showed a subsequent normal growth for the respective breeds [22,49], with no deaths reported in the first year of life.

Present results can be compared to those obtained in previous studies [14,15,16], evaluating the effect of alfaxalone on puppy outcomes at C-section. Different study design and procedures have to be considered as they make a direct comparison of the results impossible [14,15,16]. Concerning the peri-operative management of delivery, Doebeli and colleagues [14] only evaluated emergency C-sections, Metcalfe and colleagues [15] enrolled both emergency and planned C-sections, without differentiating results obtained in the two conditions, while Conde Ruiz and colleagues [16] considered only elective C-sections, using alfaxalone not only for general anesthesia induction, but also for continuous rate infusion maintenance. However, the present study results are in agreement with those reported by Doebeli and colleagues [14], that found no bias of elective or emergency conditions at C-section on the anesthetic outcome of alfaxalone on neonatal viability at birth. On the other hand, Metcalfe and colleagues [15], reported similar viability in puppies born from either elective or emergency C-sections, independently of the drug used to achieve general anesthesia (alfaxalone vs propofol), even if the evaluation of neonatal viability relied on a subjective scoring system of straightening, suction, ano-genital, and flexion reflexes, reported by the authors to be less specific than the Apgar scoring method [15]. In another study [16], a lower Apgar score was reported in association to anesthesia maintenance with alfaxalone constant rate infusion compared to isoflurane in oxygen [16]. The lower Apgar score obtained, together with a worse maternal awakening, was ascribed to the different employment of alfaxalone, while the higher intra-operative systemic blood pressure was linked to the absence of isoflurane and a lighter plan of general anesthesia [16].

Operating procedures concerning the absence of pre-medication and the delay in the administration of analgesics and NSAIDs after the extraction of the last puppy are common to mentioned literature [14,15,16] and to the present study. Thus, it cannot be excluded that the administration of different drugs at different time intervals might affect neonatal outcomes independently of the anesthetic protocol used.

As previously stated, conversely to the above mentioned studies, in which the binomial relationship between anesthesia induction protocol and the Apgar score was considered, in the present study the statistical analysis also took in consideration the cortisol concentration in amniotic and allantoic fluids, previously reported as indicators for 24 h survival prognosis [42]. In the authors’ opinion, this allows a more objective evaluation of the role of the anesthetic induction protocol and its effect on neonatal outcome.

A further consideration about the characteristics of the 2 study groups is fundamental. Conversely to previous studies performed on bitches of different breeds, sizes, ages, and parities [14,15,16], only giant-size purebred bitches were enrolled to avoid any possible confounding effect due to size-specific variations. Moreover, the statistical analysis showed the absence of differences between the two groups in terms of litter size, maternal age and parity, puppies sex ratio and birth-weight, highlighting uniformity in the studied samples. In accordance with previous reports [42], none of the above-mentioned parameter’s results were associated with fetal fluids cortisol concentrations.

Finally, alfaxalone gets benefits from its high safety, its fast clearance, and short half-life, following the quick hepatic metabolism by esterases and the very efficient renal excretion, without massive accumulations in body tissues [34,35]. Nevertheless, at term of pregnancy, the maternal hemodynamic status undergoes a physiological overload due to gestation itself, especially in giant-size dogs [52,53,54], and the low cardio-circulatory impact of alfaxalone at standard dosages (up to 3 mg/kg) [55] on the maternal side is of paramount advantage.

Some features of the use of alfaxalone at C-section still need to be verified in structured studies, meaning it would be useful to enroll and statistically compare results from elective and emergency C-sections, as well as in dogs of different breed sizes, including a wider evaluation of the maternal side. However, from a comprehensive evaluation of the literature available at present, alfaxalone can be considered as a safe drug for anesthesia management at C-section.

## 5. Conclusions

In conclusion, the present study results, obtained from healthy, viable, and normal-weighted puppies born by elective C-section performed at term in healthy giant-size purebred bitches, showed a positive statistical association between anesthesia induction protocol with alfaxalone and a higher Apgar score. Moreover, cortisol concentrations in amniotic and allantoic fluids are identified as covariates of this relationship. These findings could be a starting point for further investigations when less viable puppies (and possibly higher cortisol concentrations in fetal fluids) are detected or expected, such as at an emergency C-section.

## Figures and Tables

**Table 1:** Amniotic and allantoic cortisol concentrations measured in the 16 puppies belonging to group A and in the 20 puppies belonging to group P, expressed as mean ± SD.

	Group A (n = 16)	Group P (n = 20)
**Amniotic cortisol (ng/mL)** **(n = 36)**	7.0 ± 2.4 ^a^	6.7 ± 3.3 ^a^
**Allantoic cortisol (ng/mL)** **(n = 36)**	6.1 ± 2.7 ^a^	5.8 ± 1.9 ^a^

Equal superscripts denote the absence of statistically significant differences within columns and rows (*p* ≥ 0.05).

**Table 2:** Clinical records (neonatal gender, birth weight, Apgar score, maternal age and parity and litter-size) in group A and group P, expressed as mean ± SD. The APGAR score is expressed as a median value, with the minimum and maximum in brackets.

Clinical Records	Group A	Group P
Litters (n)	5 ^a^	5 ^a^
Puppies (n)	16 ^a^	20 ^a^
Males (n)-Females (n)	8-8 ^a^	10-10 ^a^
Birth weight (g)	664.1 ± 185.9 ^a^	794.5 ± 96.2 ^a^
Apgar score	10 (10, 10) ^a^	9 (8, 10) ^b^
Litter size (puppies n)	7.5 ± 4.2 ^a^	8.9 ± 3.4 ^a^
Maternal age (years)	2.8 ± 1.3 ^a^	4.8 ± 0.7 ^a^
Maternal parity (n)	1.4 ± 0.6 ^a^	2.0 ± 0.4 ^a^

Different superscripts denote statistically significant differences within rows (*p* < 0.05).
